# Deconstructing cardiovascular and coagulation-related traits links dietary ecology to multi-functional snake venom specificity

**DOI:** 10.1093/evolut/qpag036

**Published:** 2026-03-11

**Authors:** Matthew L Holding, M Hao Hao Pontius, Meilyn S Ward, Jaynab Akhtar, Hayley L Crowell, David Svilar, Kevin M Keeler, Laura M Haynes, David Ginsburg, Jordan A Shavit

**Affiliations:** Life Sciences Institute, University of Michigan, MI, United States; Department of Ecology and Evolutionary Biology, University of Michigan, MI, United States; Department of Pediatrics, University of Michigan, MI, United States; Life Sciences Institute, University of Michigan, MI, United States; Life Sciences Institute, University of Michigan, MI, United States; College of Pharmacy, University of Michigan, MI, United States; Department of Ecology and Evolutionary Biology, University of Michigan, MI, United States; Life Sciences Institute, University of Michigan, MI, United States; Department of Pediatrics, University of Michigan, MI, United States; Great Lakes Science Center, United States Geological Survey, MI, United States; Life Sciences Institute, University of Michigan, MI, United States; Life Sciences Institute, University of Michigan, MI, United States; Department of Pediatrics, University of Michigan, MI, United States; Department of Internal Medicine, University of Michigan, MI, United States; Department of Human Genetics, University of Michigan, MI, United States; Department of Pediatrics, University of Michigan, MI, United States; Department of Human Genetics, University of Michigan, MI, United States

**Keywords:** predator-prey, venom, hemostasis, thrombocyte, adaptation

## Abstract

Animal venoms vary greatly in compositional complexity, where complex venoms are hypothesized to be maintained by greater dietary breadth. Beyond explaining venom composition, the dietary breadth hypothesis predicts that these more complex venoms should show greater functional breadth in terms of overall toxicity and by disrupting multiple prey physiological processes. We evaluate these predictions with six distinct physiological assays of cardiac and blood clotting functions and compared the effects of venoms from snake species with a range of taxonomic dietary diversity levels. We compared the taxonomic dietary generalists *Agkistrodon piscivorus* and *Sistrurus miliarius* to taxa with varying taxonomic specialization, namely *Ag. contortrix* and *Crotalus adamanteus*, and *Azemiops feae*. Comparing fish thrombocyte and mammal platelet aggregation and fibrin clot formation, only species with broader diets disrupted both fish and mammal hemostatic function. Venom of *Ag. piscivorus*, a uniquely fish-eating species, was the most disruptive of zebrafish heart rate, thrombocyte activation, vascular permeability, and clotting after injury. *Sistrurus miliarius* was also highly toxic, whereas mammal-specialists’ venoms scarcely altered zebrafish physiology. Our results support the hypothesis that dietary breadth selects functionally complex venoms. Understanding venom gene evolution, snakebite symptoms, and searching for therapeutics in venom should be guided by evolutionary ecology.

## Introduction

Animal venoms are complex traits that function through rapid and potent pathological effects on multiple target tissues (Casewell et al., 2013), and therefore present an opportunity to study the relationship between species’ life histories and the functional complexity of their traits. Many *in vivo* assays of venom function have relied on injections into mouse models to determine LD$_{50}$ (Gibbs & Mackessy, 2009; Perez et al., 1978), complemented by *in vitro* tests on available purified or synthetic substrates (Mackessy, 2008). Extension beyond mouse models has revealed recurrent evolution of variation in potency toward different prey species (Barlow et al., 2009; Bernardoni et al., 2014; Modahl et al., 2018; Zdenek et al., 2022). This variation in potency occurs despite the general tendency of venom to target the physiological mechanisms underlying homeostasis, such as neurotransmission and hemostasis (reviewed in Casewell et al., 2013), where the protein regulators of these processes tend to be products of conserved genes and pathways (Doolittle, 2009). Cases of between-species target specificity in snake venoms therefore suggest that sufficient divergence in target proteins has occurred to select for the evolution of functional specificity (Drabeck et al., 2022; Morecroft et al., 2025).

Venomous snakes feed on a variety of vertebrate prey, with repeated evolution of increased specialization toward specific prey taxa (Grundler & Rabosky, 2021). In more generalist species, feeding on taxonomically diverse prey should favor venom that functions well across distantly related prey species. Meanwhile, a specialist might be expected to have venom that fails to function well when injected into species other than the snake’s typical prey (Barlow et al., 2009; Healy et al., 2019; Lyons et al., 2020). Previous studies have related venom composition to dietary divergence (Chijiwa et al., 2000; Daltry et al., 1996) and others found positive correlations between venom phenotypic complexity measured at the transcriptome- and proteome-level and prey taxonomic diversity in snakes (Holding et al., 2021; Schaeffer et al., 2023), snails (Phuong et al., 2016), and spiders (Pekár et al., 2018). However, it remains unclear whether and how dietary breadth maps to functional measures of toxicity across multiple tissues of diverse prey.

Vertebrate hemostasis provides an opportunity to assess multi-functional venom toxicity in a comparative framework. Hemostasis stops bleeding through blood clot formation, and can be deconstructed into its two major endpoints: primary hemostasis (the recruitment of activated thrombocytes or platelets to plug the wound) and secondary hemostasis (the reinforcement and stabilization of the clot by a fibrin mesh). Both endpoints of hemostasis are known targets of animal venoms, as are clot breakdown, vascular permeability, and overall blood pressure (Kini, 2011). The cumulative result of coagulotoxic envenomations is the widespread disregulation of hemostasis (Serrano, 2013). The breakdown of hemostasis results in rapid death of small prey, and leads to coagulopathy, swelling, and necrosis in larger potential threats to the snake, such as humans (Seifert et al., 2022). Vertebrate hemostasis is regulated largely through the coagulation cascade, a network of proteases, cofactors, and protease targets that appeared early in vertebrate evolution (Doolittle, 2009), thus facilitating comparative physiological analyses using the same methodologies across divergent species. However, the amino acid similarity of the orthologous coagulation cascade proteins can vary greatly across species, and there is evidence for functionally relevant primary sequence variation, such as the thrombin cleavage site in fibrinogen (Doolittle, 1965). Phylogenetic divergence in coagulation cascade targets could generate various forms of natural selection pressure on venoms (Holding et al., 2021; Morecroft et al., 2025).

We tested the hypothesis that greater dietary breadth selects for more functional complexity in snake venoms. To test this hypothesis, we measured multiple lethal and coagulopathic functions of venom. We first deconstructed clotting of both fish and mammal blood for comparative, *in vitro* analyses of both primary and secondary hemostasis in the presence of snake venoms. Then, we leveraged *in vivo* tests in engineered lines of larval zebrafish models to compare the effects of snake venoms on zebrafish overall survival, cardiac function, thrombocyte activation, vascular leakage, and cessation of blood loss at the site of a wound. By deconstructing both prey physiology and snake venom into their component parts, we have demonstrated how a complex prey physiological process—hemostasis—is impacted in multiple ways by venoms with different levels of efficacy.

**Figure 1.**
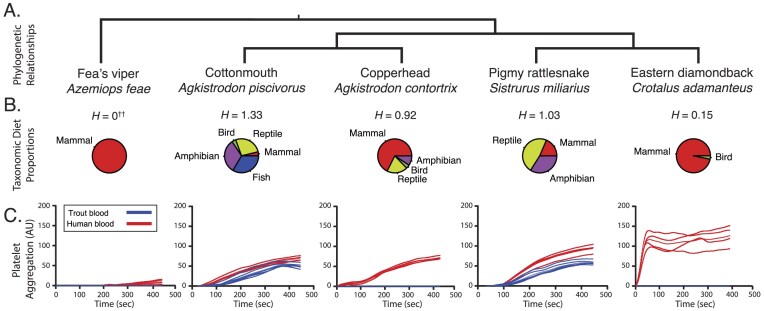
Phylogenetic relationships, dietary information, and venom effects on primary hemostasis of fish and mammal blood for five snake species. (A) Phylogenetic relationships of the five snake species. (B) Class-level taxonomic diet composition based on collated literature reports of whole prey items found in snake gut content studies and summarized to Shannon Diversity (*H*). (C) Whole blood impedence aggregometry of fish (*Salvelinus namaycush*; blue lines) and mammal blood (human; red lines) measures the activation and subsequent aggregation of thrombocytes or platelets, which are the cellular components of primary hemostasis. Only the dietary generalists’, *Ag. piscivorus* and *S. miliarius*, venoms aggregated fish thrombocytes at a detectable level, while all venoms aggregated human platelets. ^✝✝^Only a single diet record is known for *A. feae*.

We focused our experiments on the venoms from five vipers. Four are pitvipers with partially overlapping distributions in eastern North America: the cottonmouth (*Agkistrodon piscivorus*), the copperhead (*Ag. contortrix*), the pigmy rattlesnake (*Sistrurus miliarius*), and the eastern diamondback rattlesnake (*Crotalus adamanteus)*. The fifth is the southeast Asian outgroup to all pitvipers, Fea’s viper (*Azemiops feae*; Orlov et al. 2013; Figure 1A).

The four pitvipers’ diets have been quantitatively described through multiple gut content studies per species, which we collated and summed to define the vertebrate prey taxonomic preferences and taxonomic diet diversity of each snake species (Figure 1B; see Supplementary Table S1 for raw data and citations). The diet of *Ag. piscivorus* was the most taxonomically diverse, followed by *S. miliarius*, then *Ag. contortrix*, then *C. adamanteus*. The diets of two species, *Ag. piscivorus* and *S. miliarius*, did not include any one prey class as the majority of their diet, and we refer to these as generalists. Uniquely among western hemisphere vipers, fish are part of the diet of *Ag. piscivorus* (Vincent et al., 2004) and formed the most frequent prey item in our collated dataset at 33.5% of prey, followed by amphibians (32.1%). Reptiles were the most frequent prey class in *S. miliarius* (47.4%), followed by amphibians (34%). Mammals comprised the majority of vertebrate prey for *Ag. contortrix* (67.7%) and *C. adamanteus* (96.7%), and as such they show different degrees of mammalian specialization. A single report and accompanying anecdotal evidence suggests the *A. feae* outgroup species feeds on mammals. Finally, we possessed some of the above venoms in quantities too low for chromatographic purification of specific venom components, and so venom from two additional species with specialist, mammal-heavy diets, *C. molossus* (88.9% mammals) and *C. oreganus* (89.9% mammals), were used in two experiments testing fractions enriched for specific venom gene families.

## Materials and methods

### Snake venom preparation

Snake venoms were kindly provided by Dr Darin Rokyta at Florida State University. Venom samples were kept at −80 $^{\circ }$C until use. Venom was reconstituted in phosphate-buffered saline (PBS; 100 mM NaCl, 50 mM sodium phosphate, pH 7.4) to a concentration of 0.3 mg/ml for all analyses unless otherwise mentioned.

To enrich for snake venom serine proteases (SVSPs), whole venom was diluted to 20 mg/ml in HBS (25 mM HEPES, 150 mM NaCl, pH 7.4) and applied to a HiTrap Benzamidine FF(HS; Cytiva Corp.) column equilibrated with 0.05 M Tris-HCl containing 0.5 M NaCl, pH 7.4. Fractions of 0.5 mls were eluted with 0.05 M glycine, pH 3.0 and neutralized with 7.5 µL 1M Tris-HCl, pH 9. Final pH of fractions was approximately 7.4. Fractions with concentration of $>$0.150 mg/ml were pooled, treated with 1,10 phenanthroline to inhibit metalloproteinases, then buffer-exchanged into HBS.

Venom C-type lectins (CTLs) were purified using Immobilized D-galactose gel (Thermo Fisher). All flow was by gravity at 4 $^{\circ }$C. A 2 ml column volume was poured and equilibrated to PBS as binding buffer. Next, 3 ml of 20 mg/ml venom in PBS was loaded, followed by wash with 30 ml of PBS. Venom CTL was eluted with 10 ml of PBS, 0.1 M D-galactose, pH 7.2. Purified CTL was buffer exchanged into PBS by centrifugation in a 3kDA cut-off Amicon Ultra 15 ml conical filter. Both SVSP and CTL enrichments were visualized by SDS-PAGE conducted under reducing conditions on a Novex 4-20% Tris-glycine 15 well gel.

### Blood collection

Lake trout (*Salvelinus namaycush*) blood was collected under the Great Lakes Science Center ACUP Fish Euthanasia Guidelines Appendix A. Human blood was obtained from the University of Michigan Platelet Pharmacology and Physiology Core. Mouse (*Mus musculus; C57BL/6*) blood was collected under UM ACUC Protocol #PRO00012683. Blood from each species was collected using venipuncture and anticoagulated using 9:1 blood:3.2% sodium citrate. Plasma was obtained by centrifugation at 1500 x *g* for 15 min to isolate thrombocyte-poor or platelet-poor plasma, which was fresh frozen at −80 °C until use. For trout and human, a fresh whole blood portion was also kept for aggregometry.

### Aggregometry

To measure venom effects on primary hemostatsis, 300 µl of citrated trout or human whole blood was mixed with 300 µl CaCl2 in a Roche Multiplate aggregometer and allowed to incubate for 2 min. Then, 20 µl of 0.3 mg/ml snake venom, PBS, or another agonist was added to induce thrombocyte or platelet aggregation. The multiplate system measures aggregation units based on electrical impedance as thrombocytes or platelets stick to electrodes, and we took these measurements over 7 min. We extracted the highest rate of increase in aggregation units across a 20-s measurement (Vmax), the time of Vmax, the absolute maximum aggregation units reached, and the time of maximum aggregation in base R using the lm and max functions.

### Fibrin clot formation

We quantified fibrin clot formation using citrated plasma. Blood from lake trout, mice *Mus musculus*, or human subjects was collected into 3.2% sodium citrate at a 1:10 ratio, and the resulting blood was centrifuged for 10 min at 800 RCF and again at 10,000 RCF, resulting in platelet poor plasma. Fibrin clotting was initiated by adding 50 µl of HBS with 12.5 mM CaCl2 to 50 µl of plasma in a single well of a clear 96 well flat-bottom microplate. Additionally, each well contained an additional 6 µl of HBS (control) or 6 µl of 0.3 mg/ml snake venom. After recalcification, we immediately began measuring absorbance at 405 nM every 10 s for 35 min in a Molecular Devices Spectramax M2 plate reader. We used clotting and lysis tools in shiny-clot (Longstaff, 2017) to calculate clotting time and maximum absorbance for each fibrin formation curve. For statistical comparison, we extracted time of clot initiation (henceforth clot time), and final turbidity 10 min post-exposure to venom. Final turbidity is a product of total fibrin generation and fibrin fiber cross-sectional area in plasma clots (Pieters et al., 2019), which determines both the strength and elasticity of the final clot.

### Zebrafish infusion

All zebrafish work was conducted under University of Michigan ACUC #PRO00012352. ABTL zebrafish larvae were anesthetized in tricaine and mounted in 0.8% low melting point agarose on glass cover slips. The agarose around the head of the larvae was removed and replaced with water. 3 nl of 0.3 mg/ml venom or PBS with 0.1% Evan’s blue dye were infused intravenously via the pericardial vessel using pulled capillary micropipettes. We used 3- or 5-days post fertilization (dpf) larvae for experiments, which are typically 3.5 and 3.9 mm total body length, respectively (Westerfield, 2000).

### Survival study

Heart rate and presence of blood flow were measured prior to infusion of venom and every 30 min post infusion for 2 hr in 3 dpf zebrafish larvae. We analyzed heart rate using repeated-measures ANOVA (RM-ANOVA) using the anova_test function from r-statix (Kassambara, 2023) in R (R Core Team, 2021). Heart rate was set as the dependent variable, treatment as the between-subjects factor, time as the within-subjects factor, and fish ID as the subject identifier. To analyze the loss of blood flow, we used the survfit function in the survival package (Therneua, 2024) to fit Kaplan–Meier curves to the loss of blood flow in each treatment group and derive *p*-values from the log rank test comparing each curve.

### Thrombocyte adherance

To quantitatively compare the ability of each venom to activate thrombocytes, we quantified the percent change in thrombocytes attached to blood vessel walls (a marker of activation). We did so by comparing the pre-injection and post-injection number of immobile fluorescent cells in one-second video-recorded observations. Specifically, 5 dpf cd41-GFP transgenic (Lin et al., 2005) zebrafish larvae were infused with 3 nl of 0.1 mg/ml venom with 0.1% Evan’s blue dye. Larvae were imaged at 80× magnification through a GFP2 filter. A 1-min video was taken of identical regions of the caudal region of the zebrafish larvae prior to envenomation and 15 min post envenomation as previously described (Huarng & Shavit 2015). We used a one-way ANOVA to assess the effect of treatment on % change in the number of attached thrombocytes and used Tukey’s post hoc test to compare each infusion treatment to uninjected control fish.

### Vascular leakage

To quantify venom-induced vascular leakage, 3 dpf zebrafish larvae were infused with 3 nl of 0.3 mg/ml venom along with 0.1% Evan’s blue dye. The dye leaks out of damaged vessels and produces extravascular fluorescence. Fluorescence images were captured at 0, 15, and 30 min post-infusion on an Olympus IX73 at 80× magnification through an mCherry filter. We then placed seven 40 × 20 pixel regions of interest (ROI) onto each image. One ROI was placed at the bottom left corner of the image to capture background fluorescence, and then three pairs of ROI’s were placed on the focal fish, one inside the posterior cardinal vein and one immediately ventral to the vein (above extravascular tissue). We used ImageJ to export the histogram of red pixel intensities, rescaled them from 0 to 100 based on red intensity of the background ROI, and calculated a ratio by dividing mean redness inside the vessel by mean redness outside the vessel. The mean of the three resulting ratios was then taken, and divided by the mean ratio at time zero to yield our input for statistical analyses: normalized extravascular red fluorescence. We analyzed these data with a RM-ANOVA with main effects of treatment and time using the aov function in R and used pairwise t-tests for post hoc comparisons of treatment to controls.

### Laser-mediated vascular injury

To quantify venom effects on clotting at the site of a wound, laser-mediated vascular injury was performed on 3 dpf zebrafish larvae. A laser (MicroPoint Pulsed Laser System, Andor Technology, Belfast, Northern Ireland) was used to injure the posterior cardinal vein (PCV) 5 somites caudal to the anal pore using 99 pulses. The venom samples used were specifically enriched, via chromatographic separation, for snake venom serine proteases (SVSP, Figure 6A; Supplementary Figure S3). Venom SVSP are the best described class of snake venom protein in terms of coagulotoxic function (Kini, 2011) and we wished to determine if signals of fish-specificity could be extended specifically to the SVSPs. We measured time to occlusion of the vessel (via a blood clot) in a 120 s video, and compared the *Ag. piscivorus* venom fraction to other mammal-specialist snake species from the genus *Crotalus* (Supplementary Table S1), as well as to *S. miliarius*. Unfortunately, the isolated *S. miliarius* SVSPs resulted in rapid cessation of fish heartbeat, preventing quantitative evaluation of the interaction between SVSP-enriched venom and vein ablation. Venom from this species was accordingly excluded from this experiment. We used a one-way ANOVA and Tukey’s post hoc test to assess the effect of treatment on wound occlusion time, and a Chi-square test to compare the proportion of occluded wounds after one hour.

## Results

### Comparative coagulotoxic effects in fish vs. mammal blood

Venoms disrupt primary hemostasis by activating of thrombocytes (in non-mammalian vertebrates) or platelets (in mammals; Kini, 2011), which reduces the availability of these cells for clot formation at the bite wound or sites of venom-induced hemorrhage (Drabeck et al., 2022; Thomazini et al., 2021). To assess functional breadth of venoms as effectors of primary hemostasis, we compared each venom’s effects on aggregation of trout thrombocytes and human platelets. Significant aggregation in trout blood was only caused by *Ag. piscivorus* and *S. miliarius* venom (Figure 1). Meanwhile, human blood samples aggregated measurably in response to all venoms tested, but to varying extents (Supplementary Table S2). The venom of *A. feae* caused the lowest detectable human blood aggregation, followed by the venoms of *Ag. piscivorus, Ag. contortrix*, and *S. miliarius*. The venom of the mammalian specialist *C. adamanteus* resulted in the fastest and greatest degree of human blood aggregation among the venoms tested. Aggregation of both fish thrombocytes and mammalian platelets by only the venoms from snakes with the most diverse diets supports the hypothesized association between dietary diversity and venom functional complexity.

**Figure 2.**
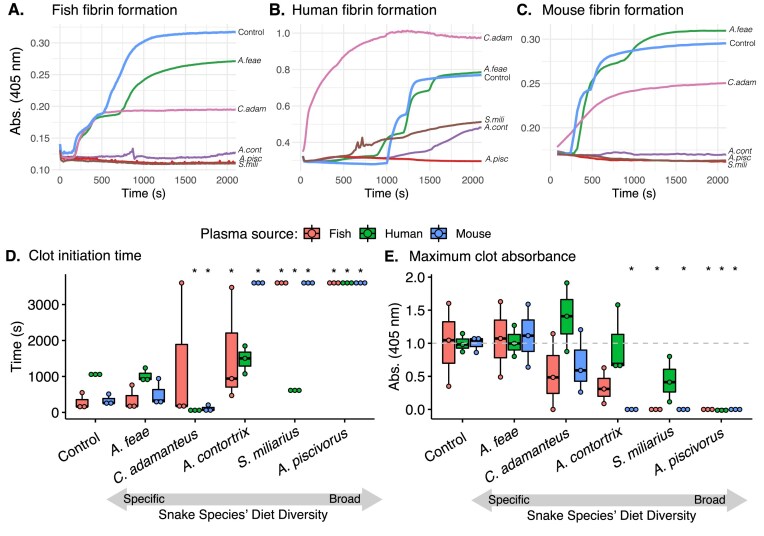
Effects of five snake venoms on two secondary hemostasis phenotypes in fish and mammal blood. Thrombocyte-poor or platelet-poor plasma from fish *S. namaycush*, human, or mouse *Mus musculus* were incubated with either saline control or 0.3 mg/ml venom from *A. feae, Ag. piscivorus, Ag. contortrix, S. miliarius*, or *C. adamanteus* and recalcified to permit fibrin clot formation. Snake species are ordered from left to right by the Shannon Diversity value of their class-level diets. Sample absorbance was measured at 405 nm to track fibrin formation dynamics. Representative profiles of (A) fish, (B) human, and (C) mouse fibrin formation show differential impacts of venom on the initiation and maximum absorbance of fibrin clots. We extracted two phenotypes from the fibrin formation curves: (D) clot initiation time and (E) maximum clot absorbance (a measure of clot structure). Asterisks indicate significant differences between venom-treated and saline controls for a given plasma type. The dashed horizontal line in panel E represents mean max absorbance value of each plasma in the presence of only saline control (normalized to 1.0).

Venoms disrupt secondary hemostasis by either accelerating or inhibiting the conversion of fibrinogen to fibrin (Kini, 2011). Accelerated fibrin formation can produce occlusive clots that block blood flow to organs, while inhibition aids in venom-induced hemorrhage by preventing wound closure (Seneci et al., 2021b). To investigate direct venom effects on secondary hemostasis, we measured venom-induced changes to fibrin clot formation in trout, human, and mouse plasma. Venom had differential impacts on the trajectory of fibrin clot formation (Figure 2A–C). In fish plasma, *Ag. piscivorus* and *S. miliarius* venoms completely prevented clot formation, and hence clot times were significantly longer and turbidities significantly lower than baseline controls. These venoms had similar anticoagulant effects on mouse plasma, while only *Ag. piscivorus* maintained significant anticoagulant effects on human plasma (Figure 2D,E). In human plasma, *S. miliarius* became procoagulant by significantly decreasing clot time. Among mammal-feeding snakes, only *Ag. contortrix* produced a significant effect on fish plasma by extending clot time without inducing statistically significant effects on final clot turbidity. Venom of *Ag. contortrix* prevented clotting altogether in mouse plasma, but had no significant effects on human plasma clotting. *Crotalus adamanteus* venom showed strong procoagulant tendencies in mammalian fibrin clotting, where we saw significant decreases in clot time of both human and mouse plasma in the presence of this snake’s venom. Finally, the venom of *A. feae* was largely ineffective at inducing significant changes to plasma clotting parameters in fish and mammalian samples. Notably, only *Ag. piscivorus* and *S. miliarius* significantly changed the clot time of all three plasma types tested, and only *Ag. piscivorus* venom produced significant changes in final turbidity of all plasmas, consistent with the hypothesized relationship between venom functional breadth and more diverse diets.

Next, we experimentally tested for differential effects of each venom on several independent cardiac, vascular, and coagulation-related phenotypes in zebrafish, predicting that venoms from the dietary generalists would enact the most toxic effects in the independent assays, and that *Ag. piscivorus* would be most effective as a species that feeds heavily on fish in addition to other vertebrate groups.

### Cessation of zebrafish cardiac function

**Figure 3.**
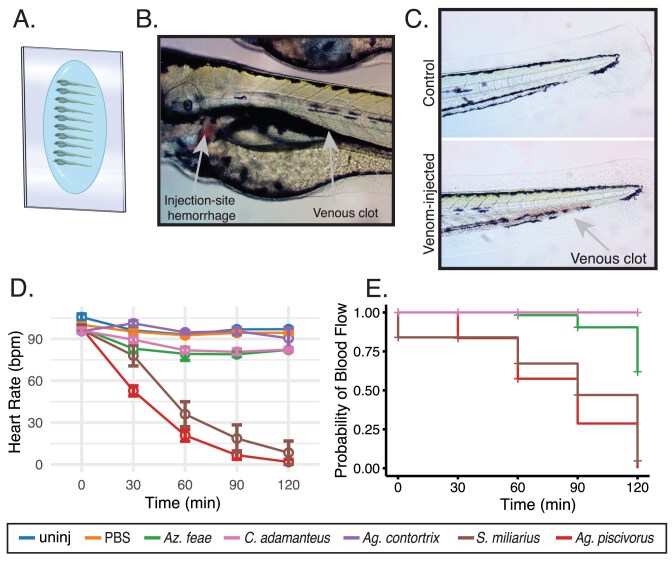
Effects of five snake venoms on two zebrafish cardiovascular phenotypes: heart rate and blood flow. (A) Zebrafish at 3 dpf (in a cartoonized depiction) were arranged onto microscope slides in groups of ten fish each and either left uninjected (control) or injected in the pericardial vessel with 3 nl of either PBS (control) or 0.3 mg/ml venom from *A. feae, Ag. piscivorus, Ag. contortrix, S. miliarius*, or *C. adamanteus*. Observation of control and envenomated fish showed that venom-induced phenotypes included (B) injection-site hemorrhage, clots in the venous return just caudal to the heart and swim bladder, as well as (C) clots in the caudal vein just after the junction with the caudal artery at the tip of the tail. Immediately post-injection and in four subsequent 30 min intervals, we (D) measured each fish’s heart rate by counting beats for 20 s. Error bars are 1 standard error of the mean. (E) We also recorded whether or not blood flow was observable and produced survival curves depicting the loss in blood flow in each treatment group. Species names are entered in the legend in order of increasing class-level dietary diversity, from left to right.

Loss of heartbeat and blood flow are clear markers of prey death, and therefore successful envenomation by the snake. Injection of *Ag. piscivorus* venom into larval zebrafish (Figure 3A) produced several observable phenotypes, including apparent hemorrhage at the venom injection site and aggregations of red blood cells indicative of a clot. Apparent clots were common near the heart (Figure 3B) and in the tail where the caudal vein and artery join (Figure 3C). Blood flow and heartbeat—the two hemostasis-related vital signs we tracked—were partially uncoupled; we often observed the latter without observable flow, presumably due to clot formation.

The time course of loss in both heart rate and blood flow by zebrafish differed among venoms. Overall, venom slowed zebrafish heart rate over the 120 min observation period (repeated-measures ANOVA, *F* = 62.3, *p*$<$ 0.0001). At 30 min post injection, *Ag. piscivorus* was the only venom to significantly lower heart rate compared to both uninjected and PBS-injected controls (*p*$<$ 0.001, Figure 3D, Supplementary Figure S1). Sixty minutes post-exposure, *S. miliarius* venom had also reduced heart rate compared to controls (*p*$<$ 0.01). Both *Ag. piscivorus* and *S. miliarius* venom continued to reduce heart rate over the next hour and fish injected with these two venoms did not differ significantly in heart rate beyond the initial 30 min observation. Meanwhile, the venoms of the three species with a majority mammalian diet yielded limited (*A. feae* and *C. adamanteus*) or no reduction (*Ag. contortrix*) in heart rate. See Supplementary Figure S1 for all post hoc pairwise comparisons for heart rate. The proportion of fish with observable blood flow decreased significantly relative to the controls in the group treated with *Ag. piscivorus* and *S. miliarius* venom (Figure 3 D, *p*$<$ 0.001), and blood flow reduction did not differ significant between these two venoms (*p* = 0.13). The venoms of snakes with more mammal-specialist diets were much less effective, with only modest losses in the *A. feae*-venom-injected cohort (*p*$<$ 0.001) and no observed loss of blood flow in fish injected with *Ag. contortrix* or *C. adamanteus* venom.

### Effects on zebrafish thrombocyte function

**Figure 4.**
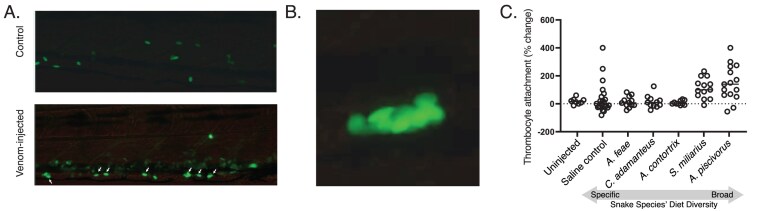
Differential effects of five snake venoms on zebrafish thrombocyte activation, a key phenotype for perturbed primary hemostasis. Zebrafish with GFP-tagged thrombocytes (green fluorescent cells) were injected at age 5 dpf with 3 nl of 0.3 mg/ml venom from *A. feae, Ag. piscivorus, Ag. contortrix, S. miliarius*, or *C. adamanteus*. Snake species are ordered from left to right by the Shannon Diversity value of their class-level diets. After 30 min, we observed signs of thrombocyte activation based on (A) elongation and vessel wall attachment (white arrows) and (B) clumped aggregates of multiple cells. (C) Thrombocyte activation was scored based on the change in numbers of circulating fluorescent cells. Only the venom of fish-eating dietary generalist *Ag. piscivorus* significantly changed levels of thrombocyte attachment relative to uninjected and saline-injected control fish.

Primary hemostasis in fish is mediated by thrombocytes, and we sought to directly compare effects of each snake species’ venom on these cells in living fish. We used the cd41-egfp transgenic reporter line of zebrafish to visualize and track thrombocytes as they flowed through live fish vessels (Lin et al., 2005; see Supplemental Video 1). In response to venom, we observed activated thrombocytes (elongated shape and adherent to the vessel wall, Supplemental Video 2), particularly in the caudal vein on the ventral side of the fish (Figure 4A). We also observed large thrombocyte aggregates moving through fish blood vessels in response to injection with *A. piscovorus* venom specifically (Figure 4B; see Supplemental Video 3), which could be fitness-relevant if they later block circulation (i.e., embolism). Treatment had a significant effect (Figure 4C; ANOVA *F* = 6.0, *p* = 0.0003), and the results mirrored the survival analyses, with *Ag. piscivorus* venom inducing the largest mean increase in thrombocyte attachment (148%; post hoc comparison to PBS-injected fish: *p* = 0.0025). The mean change in attachment for *S. miliarius* was 111%, but this effect was not significantly different from the distribution of uninjected or PBS controls in post hoc comparison, nor that of any other venom treatment (all *p*$>$ 0.1). These results are consistent with feeding on fish as a source of selection to maintain *Ag. piscivorus*’ high potency.

Snake venom C-type lectins (CTLs) are known to cause mammalian platelet aggregation, and we isolated these proteins in an attempt to pinpoint the source of species’ differences in venom action against zebrafish thrombocytes. We isolated venom CTLs from *Ag. piscivorus* and a mammal-feeding snake species, *Crotalus molossus*. However, these experiments failed to generate observable effects in our thrombocyte attachment assay, regardless of snake species (Supplementary Figure S2), indicating that the effects of CTL (at least in isolation) are not the cause of thrombocyte aggregation observed following whole venom injection.

**Figure 5.**
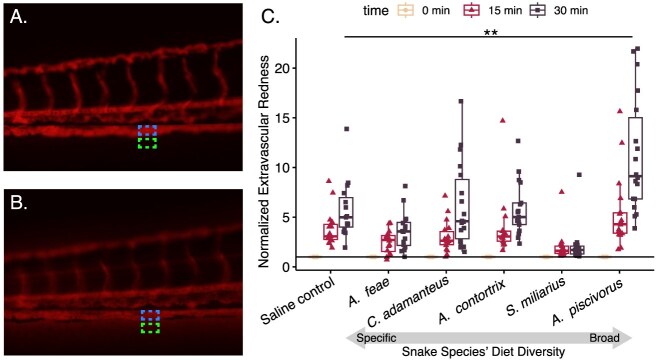
Effects of five snake venoms on vascular permeability, correlate of venom spreading and venom-induced hemorrhage. Larval zebrafish were injected intravascularly with Evan’s blue dye, which fluoresces red. The images show the same fish (A) immediately after injection and (B) 30 min later, where red color is now observable both in the vessels and also below the most ventral vessel in the fin tissue. The dashed boxes show a representative comparison of the same intravascular (blue) and extravascular (green) spaces in the in the fish. The ratio of average redness within and just below the vessels was calculated for fish injected with PBS or 0.3 mg/ml venom from *A. feae, Ag. piscivorus, Ag. contortrix, S. miliarius*, or *C. adamanteus* and then normalized to the ratio at 0 min post-injection. Snake species are ordered from left to right by the Shannon Diversity value of their class-level diets. ** indicates *p*$<$ 0.01.

### Effects on zebrafish vascular leakage

Induction of vascular permeability is crucial to the lethality of venoms, as open vasculature allows venom components to spread rapidly beyond the site of the bite. To determine whether *Ag. piscivorus* venom would be most effective at permeabilizing fish vasculature, we compared snake venoms in a zebrafish model of vascular leakage by co-injecting red dye and venom (Figure 5A, B). Over a 30 min observation period, the ratio of intravascular to extravascular red fluorescence increased, including in the PBS-injected control. *A. piscivorus* venom was the only treatment to yield a significantly higher degree of extravascular fluorescent dye than the PBS control by the end of observation, supporting the hypothesis that it is adapted to permeabilize the fish vasculature (Figure 5C).

### Effects on zebrafish wound occlusion

**Figure 6.**
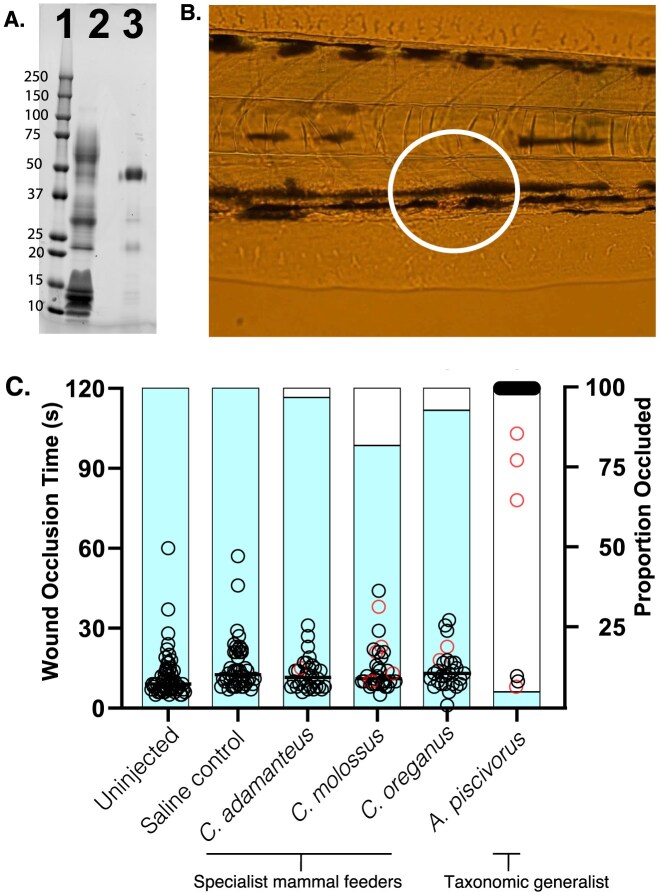
Effects of serine protease fractions of four snake venoms on the cessation of blood flow in a fresh wound. (A) Snake venom serine proteases (SVSP) were enriched with benzamidine sepharose affinity chromatography and visualized on SDS-PAGE. Lane 1: Molecular weight ladder; Lane 2: Whole *Ag. piscivorus* venom; Lane 3: Benzamidine sepharose column eluate showing bands between 20 and 50 kDA, the size of differentially glycosylated SVSP. (B) Zebrafish were injected with SVSPs and then subjected to laser injury of the posterior cardinal vein. Normally, a clot quickly occludes the vessel, visible on the image within the white circle. (C) SVSPs from four snakes were tested: the rattlesnakes, *C. adamanteus, C. molossus*, and *C. oreganus* that largely specialize on mammal prey, as well as the fish-eating dietary generalist *Ag. piscivorus*. Compared to uninjected and saline-injected fish, time to wound occlusion via blood clot was significantly increased by treatment with *Ag. piscivorus* venom only. Snake species are ordered from left to right by the Shannon Diversity value of their class-level diets. Circles indicate occlusion time for each fish, with black circles showing wounds that remained occluded, whereas red circles indicated that the blood clot broke down allowing blow flow to resume. The color fill inside the bars indicates the overall proportion of wounds that were found to be occluded one hour post-infusion (right side *y*-axis).

Anti-coagulation and associated bleeding at the bite site and at secondary sites of blood vessel injury serves to weaken bitten prey. We compared isolated SVSPs from four species’ venoms (Supplementary Figure S3) in our zebrafish *in vivo* wound occlusion model, measuring the time to wound closure via a blood clot (Figure 6B). Venom treatment was a significant predictor of time to wound occlusion (*F* = 290.144, *p*$<$ 0.0001). The significant effect of venom was attributable to SVSP from *Ag. piscivorus*, which resulted in significantly increased vessel occlusion times compared to uninjected and PBS controls as well as all *Crotalus sp*. venoms (all post hoc test *p*$<$ 0.0001). Moreover, occlusion time in fish injected with the three *Crotalus sp*. venoms did not differ significantly from controls (*p*$>$ 0.5; Figure 6C). Occlusion only occurred in 6 of 41 fish that received *Ag. piscivorus* venom, and four of those clots failed and blood flow began again before the end of 120 s observation, suggesting they were unstable clots. Meanwhile, occlusion was achieved within the 120 s observation period in 100% of fish in both control groups and in those infused with *Crotalus sp*. venoms. All wounds in the uninjected and PBS-infused controls were still occluded after 1 hr. The distribution of non-occluded wounds after one hour differed from random expectations (Chi-square goodness of fit: $\chi ^{2}=36.4$, df = 5, *p*$<$ 0.0001) and post hoc two-sample proportion tests comparing PBS treatment to each venom treatment revealed significantly lower proportions of occluded wounds in *Ag. piscivorus*-treated fish (*p*$<$ 0.0001), and in *C. molossus*-treated fish (*p* = 0.012)

## Discussion

By deconstructing the cardiovascular and clotting phenotypes, we have provided functional evidence linking venom adaptation to the diversity of prey consumed by a given snake species. Venoms from *Ag. piscivorus* and *S. miliarius*, which had the most generalist diets, showed clear efficacy in disrupting normal function in multiple independent assays of fish physiology while maintaining potency against mammalian platelet and fibrin clotting. On the other hand, functional toxicity toward only mammals occurred in concert with the more specialized, mammal-heavy diets of *Ag. contortrix, Crotalus adamanteus*, and *A. feae*. Our work highlights how a venomous species’ ecology can predict the presence of enzymes with novel functions, and demonstrates the utility of zebrafish as a model for the dissection of the complex molecular biology of envenomation.

Our work revealed signatures of adaptive function concordant with known differences in dietary breadth among snakes (Grundler & Rabosky, 2021; Holding et al., 2022). Like many prior investigations of snake venom, we included a direct link to venom lethality (Gibbs & Mackessy, 2009) by quantifying overall heart rate and blood flow. In these tests, the fish-eating species enacted the fastest drop in heart rate in the focal zebrafish model. We then deconstructed toxic functions to reveal how only the dietary generalists, *Ag. piscivorus* and *S. miliarius* (Smiley-Walters et al., 2017) produced significant effects on independent *in vivo* and *ex vivo* tests of fish thrombocyte aggregation, fish fibrin clotting parameters, and the degree of metalloproteinase-driven vascular leakiness. Both *Ag. piscivorus* and *S. miliarius* are true taxonomic generalists, with amphibians, reptiles, and mammals contributing heavily to both species’ diets and *Ag. piscivorus* adding fish (Royal & Farrell, 2021; Vincent et al., 2004). In turn, these two species also enacted potent physiological perturbations to mammalian platelet aggregation and fibrin formation. The other viper species tested, all of which possess a mammal-heavy diet, aggregated only mammalian platelets. In fibrin clotting, *C. adamanteus* showed strong procoagulant activity against mammalian plasmas, but reduced activity against fish plasma. Venom from *Ag. contortrix* more modestly extended fish fibrin clotting time, while showing major anticoagulant effects in mouse, but not human samples. As patterns of functional specialism and generalism extended to multiple distinct functions of venom, our results provide a mechanistic insight into the sources of selection acting on the unlinked, multi-gene families comprising venom. Venom proteomes from diverse animals tend to be more complex in composition when a greater taxonomic diversity of prey are consumed (Holding et al., 2021; Pekár et al., 2018; Phuong et al., 2016; Schaeffer et al., 2023). We suggest that the link between phylogenetically-diverse diets and complex venoms (Holding et al., 2021; Schaeffer et al., 2023) involves selection for breadth of potency.

We were able to document prey-specific venom function on both primary and secondary hemostasis. Prey-specific coagulotoxic venom function has been previously reported in venoms from several vipers, including *Bothrocophias microphthalmus, Bothrops lojanus, Daboia russelli* and several rattlesnakes (Bourke et al., 2025; Seneci et al., 2021a; Zdenek et al., 2022). For example, several recent studies have compared diverse venom effects on clot time and clot strength of amphibians, bird, reptile, and mammal plasmas, often revealing species-specific effects concordant with dietary preference (Youngman et al., 2022). Moreover, some metalloproteinase paralogs produced in the same snake species’ venom have been shown to enact procoagulant effects in a species-specific manner (Bernardoni et al., 2014), suggesting that generalist venoms are the result of duplicated, subfunctionalized venom genes. We extend this previous work by testing coagulotoxic function *in vivo* and *ex vivo* in fish, where dietary breadth and toxicity again largely correspond. Moreover, our cd41-GFP fish revealed a distinct effect of *Ag. piscivorus* venom on thrombocyte aggregation in the vessels, including adherent thrombocytes and free-floating multi-cell thrombi. These effects are negligible or absent in all species whose primary diet consists of mammals. Secondary hemostasis, on the other hand, showed contrasting, venom specific effects consistent with prior work in other snake and prey species, but here there were consistent, potent prevention of clot formation in fish plasma for only those snake species that eat largely non-mammal prey. These results were bolstered by the outcome of our laser wounding model, where *Ag. piscivorus* venom consistently prevented wound closure in comparison to both the strongly (mammalian-) procoagulant *C. adamanteus* and two additional mammal-feeding species not included in the other experiments (*C. molossus* and *C. oreganus*).

Our work extends the detection of differential coagulotoxic effects of venom to comparisons among mammal species, and therefore underscores the degree of specificity that is possible in venom-target interactions. While venoms tended toward either procoagulant or anticoagulant function on both human and mouse plasmas, the venom of *S. miliarius* was procoagulant on human plasma but strongly anticoagulant with mouse plasma. Similarly, the many-horned viper *Bitis caudalis* showed procoagulant effects on amphibian and lizard plasma but anticoagulant effects on bird and mammal plasma (Youngman et al., 2022). These contrasting results may reflect the nature of hemostasis as a balance between clotting and fibrinolysis (Chapin & Hajjar, 2015); a balance which may be driven in one direction or another depending on different binding affinities or protease cleavage site sequences in homologous targets of venom in different prey species. Investigations of individual, closely related prey clades or even closely related species may reveal how fine-scaled the differential effects of venom can be (Biardi, 2008), and lead to discovery of the molecular basis of specificity through comparison of amino acid sequences among species. Our results also underscore how murine models can be poor predictors of the pathology of human envenomations (e.g., Silva et al. (2022), supporting a multi-model approach to the study of this topic.

We assessed both vascular permeabilizing function (an SVMP-linked function) and the prevention of clot-based wound closures (using SVSP enriched venom fractions) *in vivo*. SVMPs and SVSPs are the most abundant venom components (and often the largest paralogous venom gene families) for most vipers (Hogan et al., 2024; Mackessy, 2008). Our assays therefore deconstruct these important venom components to study their functional specificity. The SVSP-enriched fractions from *Ag. piscivorus* were highly effective at preventing zebrafish from forming a clot at the site of injury, while its whole venom was the only one to produce a detectable increase in vascular permeability. In the wild, a fish bitten by *Ag. piscivorus* may bleed more at the bite site, and as venom metalloproteinases destroy vessel walls, those gaps would also be less able to heal.

We also isolated and tested venom CTL specifically, but with no observable effect on fish. Venom CTL from *Lachesis muta* did show cardiotoxic effects on larval zebrafish, but rather than injection, fish were incubated in water containing 80 mg/ml CTL (Zanotty et al., 2019). Thus, our results are not directly comparable to this previous work. The effects of venom CTL on thrombocyte aggregation are either synergistic with other venom components, CTL from the snakes studied here was not tested in high enough concentrations to be effective, or CTLs had effects which we simply did not observe.

Snake dietary ecology and evolutionary relationships guided our choice of focal venoms for this study. Mammals are estimated to form the majority of ancestral pitviper diets (Grundler & Rabosky, 2021), while fish-feeding is clearly a derived trait, as is feeding more on frogs than on mammals. Therefore, a parsimonious scenario for the evolution of fish thrombocyte aggregating venom may be proposed: aggregatory function may have been gained independently in both *A. piscvorus* and *S. miliarius*, in concert with taxonomic broadening of their diets to include fish and/or frogs.

Comparative models are crucial to gain a greater understanding of venom function, particularly considering both taxonomic venom specificity and evolved venom resistance in certain prey species (Healy et al., 2019; Holding et al., 2016a; Lyons et al., 2020; Perez et al., 1978). Our work here demonstrates the utility of the larval zebrafish as a comparative model for multi-functional studies of venom toxicity as well as comparative evolutionary biology (Sofyantoro et al., 2024). Zebrafish were previously employed in four studies of snake venom: to investigate histological and pathological effects of a single snake venom (Carvalho et al., 2018; Sofyantoro et al., 2024), define cardiotoxic effects of a single venom CTL (Zanotty et al., 2019), and as a survival model to demonstrate protective effects of human tryptase against venom components (Anderson et al., 2018). Additionally, zebrafish have been effectively employed in behavioral assays of the bioactive and neurotoxic effects of marine invertebrate venoms (Bosse et al., 2021; Columbus-Shenkar et al., 2018; Himaya et al., 2015; Surm et al., 2024). We demonstrate here the usefulness of the zebrafish model in an evolutionary comparative framework to understand venom multi-functionality and multi-functional specificity. We also leveraged a genetically modified reporter line, cd41-GFP (Lin et al., 2005), which enabled real-time visualization of venom effects *in vivo* within blood vessels on a single cell type. There are hundreds of such zebrafish lines (Choe et al., 2021), and, in concert with the transparent nature of larvae and potential for large sample sizes, could yield many key insights into the pathology of envenomation for both biomedical, therapeutic, and future evolutionary studies.

## Conclusion

In conclusion, our work links dietary breadth to multiple aspects of venom function, likely through ancient differentiation in prey physiological targets. These functional results complement previous work relating venom compositional complexity to dietary breadth (Holding et al., 2021; Schaeffer et al., 2023), by confirming functional diversity in the venoms of dietary generalists. Instances of more evolutionarily recent prey switching among snake species (e.g., Chijiwa et al., 2000; Martins et al., 2002; Shine et al., 2002) should be examined as natural tests of the potential for convergent evolution of venom toxins and of how quickly taxon-specific venoms emerge. Although coevolution with venom-resistant enemy species has garnered significant attention in animal venom research (Casewell et al., 2013; Holding et al., 2016b), some portion of venom evolution can be attributed to taxonomic affinities independent of coevolution. The generation and maintenance of variably complex venoms likely reflects a combination of taxonomic dietary diversity and coevolution, which future studies should seek to elucidate.

## Supplementary Material

qpag036_Supplemental_Files

## Data Availability

All data are public and available on University of Michigan—Deep Blue Data—https://doi.org/10.7302/49t4-k735.
