# Supplementary material for: Deconstructing cardiovascular and coagulation-related traits links dietary ecology to multi-functional snake venom specificity
Source: Evolution. 2026 Mar 11;80(5):1035–46. doi: 10.1093/evolut/qpag036 (PMC13168819; doi:10.1093/evolut/qpag036)

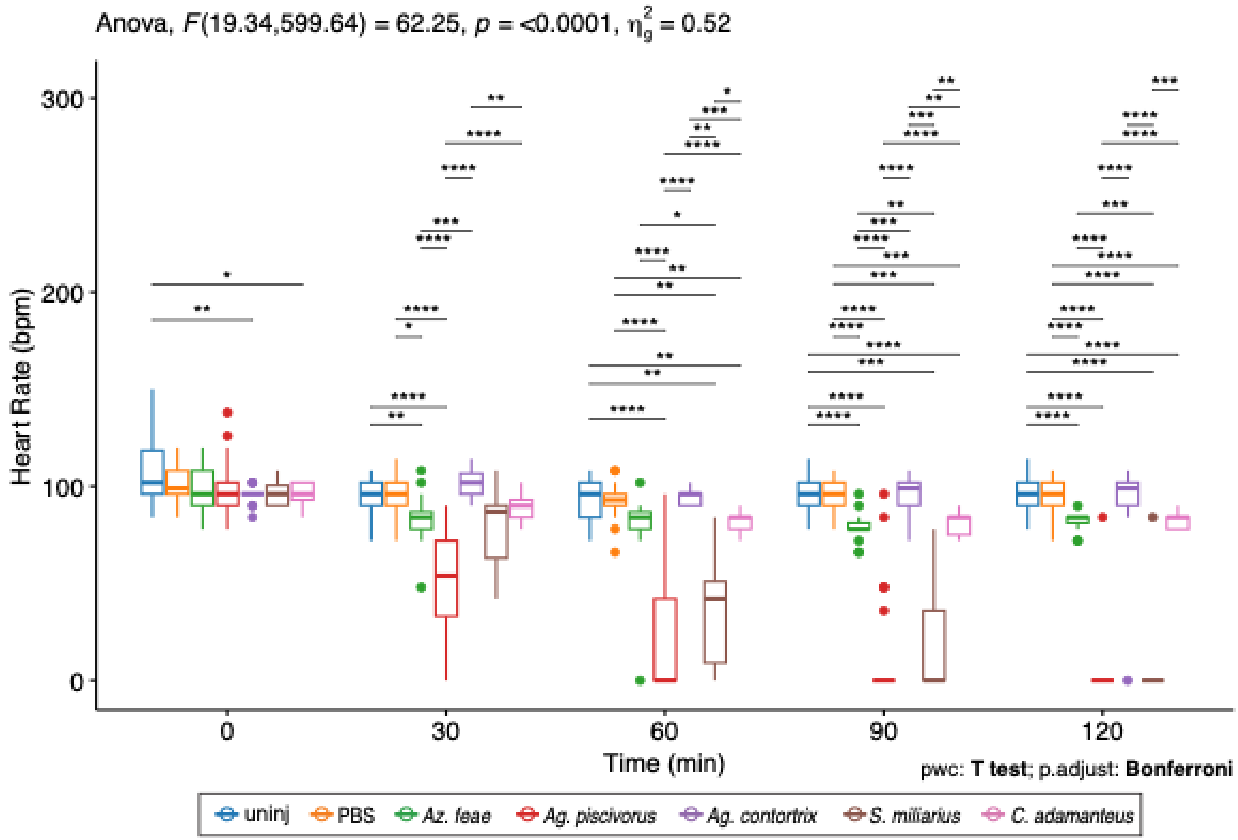

Supplemental Figure S1: Effects of venom treatment on zebrafish heart rate. 3dpf larval zebrafish were uninjected (control), PBS-infused (control) or infused with 3nL of 0.3mg/mL venom from one of five snake species. Boxplots show the heart rate over a 20 second observation at each of 5 timepoints post-injection. Lines above boxes represent all pairwise post hoc comparisons based on two-sample t-tests and Bonferroni-corrected p-values. *P < 0.05; **P < 0.01; ***P <$0.001; ****P < 0.0001

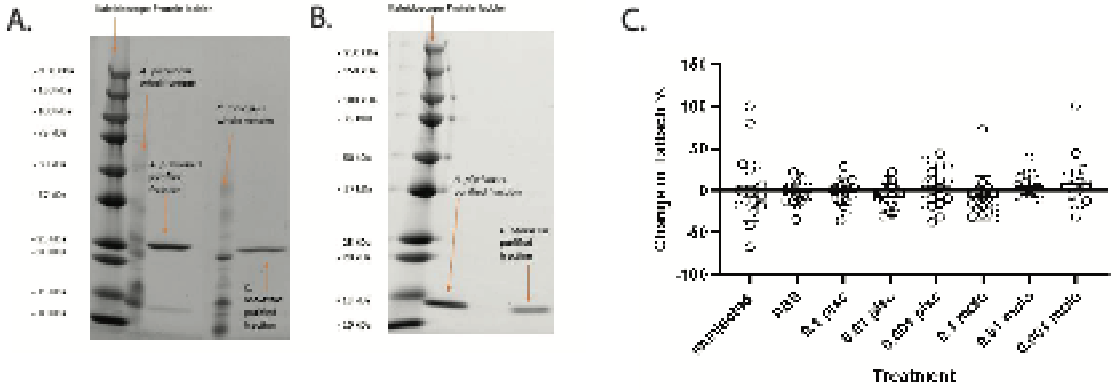

Supplemental Figure S2: Purified venom C-type lectins did not produce observable thrombocyte phenotypes. A) Non-reducing and B) reducing SDS-PAGE was used to visualize purified CTL fractions for *Agkistrodon piscivorus* and *Crotalus molossus*. 5d.p.f. CD41-GFP zebrafish larvae were injected with 3nL of 0.001, 0.01, or 0.1mg/mL purified CTL. Thrombocyte movement was recorded before and after infusion of PBS or venom, and C) the percent change in mobile thrombocytes is shown. There were no observable differences between uninjected, PBS, and venom-treated fish ($P > 0.2$).

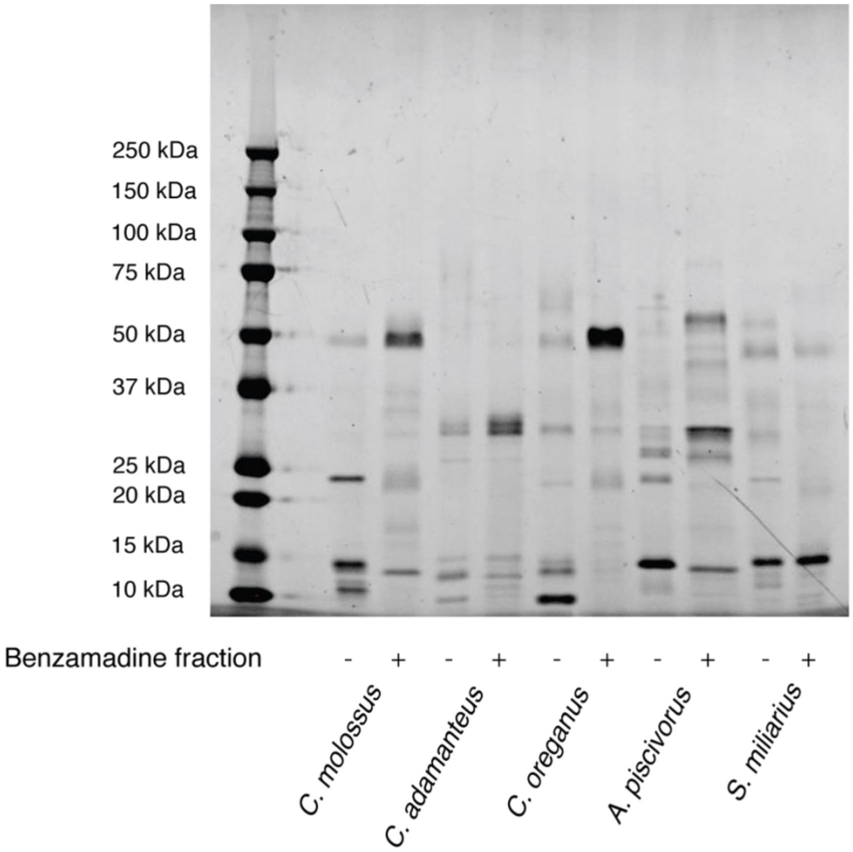

Supplemental Figure S3: Reducing SDS-PAGE was used to compare the composition of *Crotalus molossus*, *Crotalus adamanteus*, *Crotalus oreganus*, *Agkistrodon piscivorus*, and *Sistrurus miliarius* venoms before (-) and after (+) benzamidine sepharose column purification. Venoms show enrichment of certain bands between 20 and 50 kDA, consistent with successful enrichment of differentially glycosylated SVSP isoforms. The leftmost lane contains Kaleidoscope protein standards (BioRad, cat #1610375).

Supplemental videos showing cd41-egfp zebrafish. Thrombocytes and thrombocyte progenitors in the tissue glow green.

5 day old fish -- cd41-egfp Tag -- 3 nL of HBS or 0.3 mg/mL *Agkistrodon piscivorus* venom

Supplemental Video 1: Control Fish injected with HBS only. Ventrum of fish is at bottom, head to the right, and tail to the left

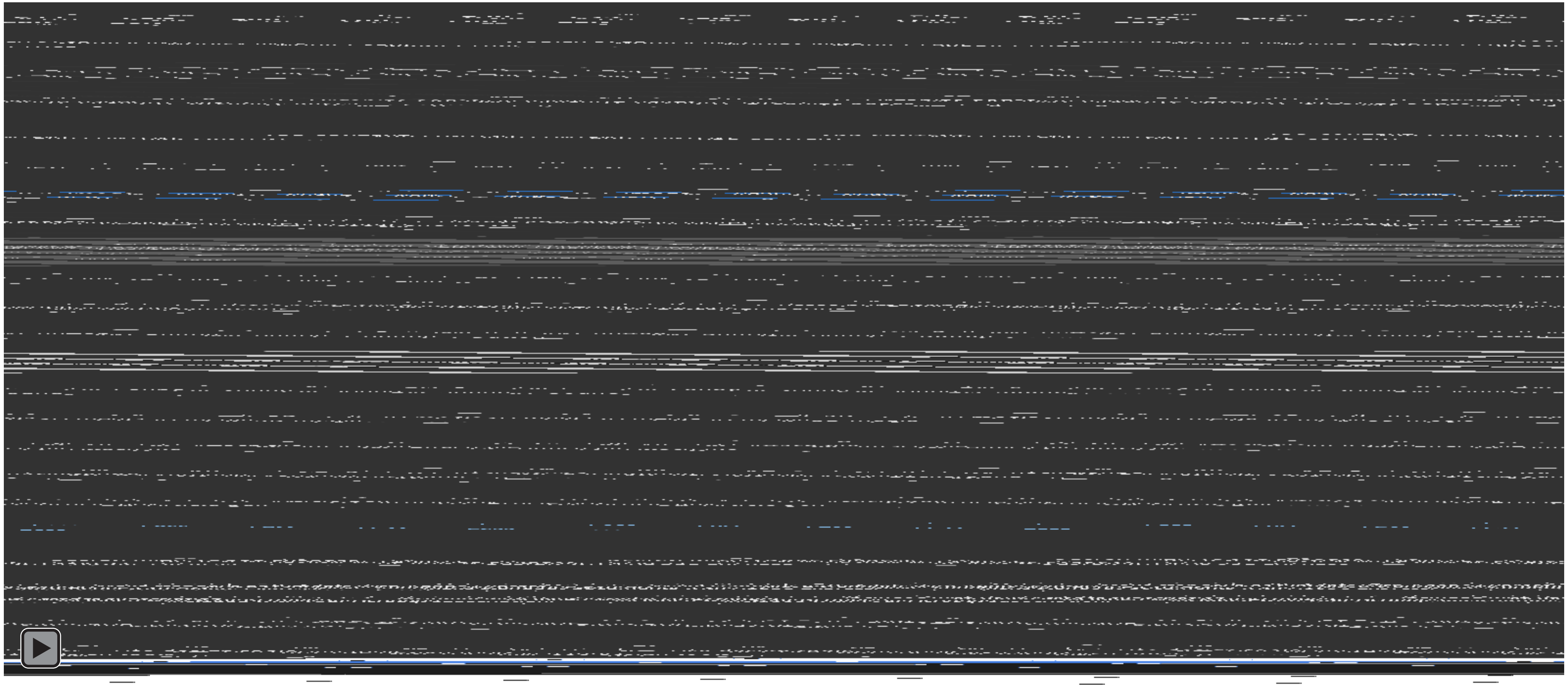

Supplemental Video 2: *A. piscivorus* venom caused thrombocyte activation (elongated green cells) and vessel adherence (cells that are either completely stationary in ventral vein at bottom of image or slowly rolling along the vessel wall

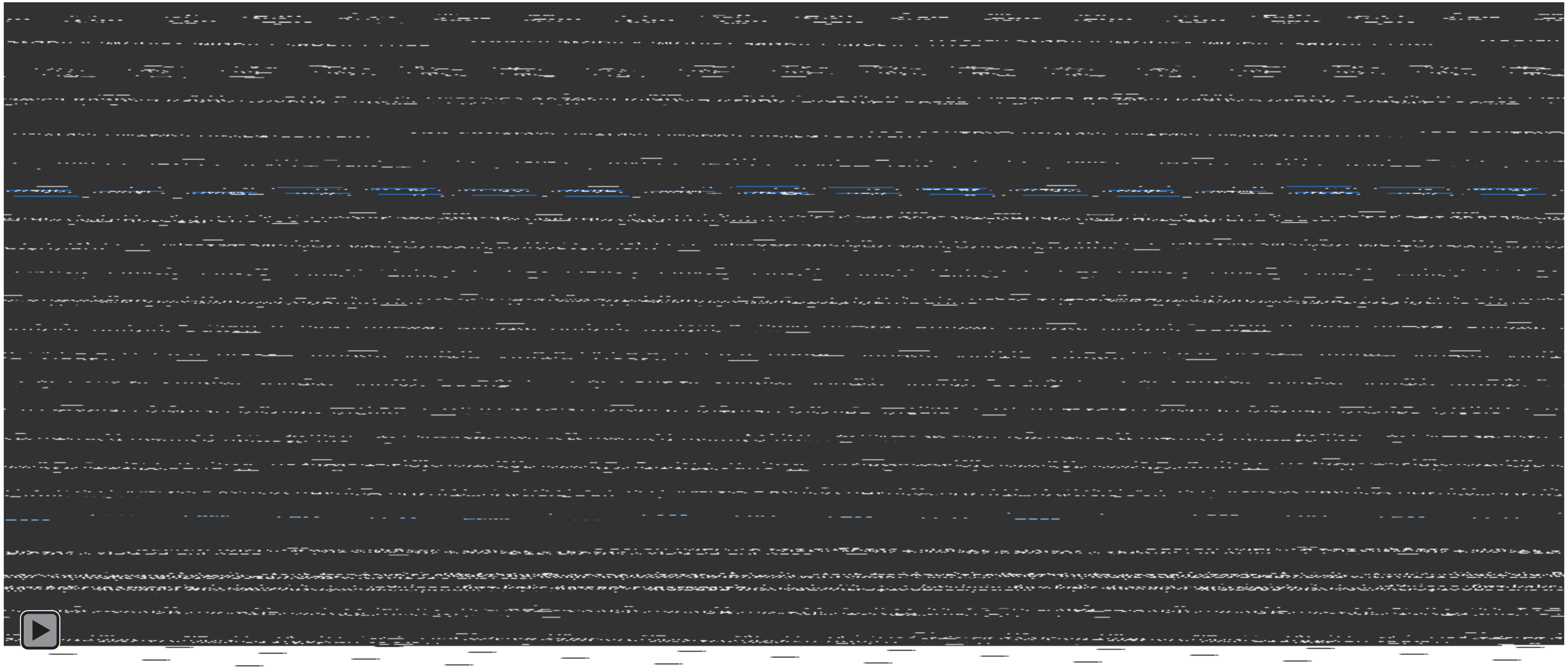

Supplemental Video 3: Individual, mobile thrombocytes have largely been cleared from circulation after 30 minute incubation with *A. piscivorus* venom, except a few large aggregates (thrombi) composed of multiple thrombocytes adhered to one another, as can be seen in this video (~10 thrombocytes in a mobile aggregation).

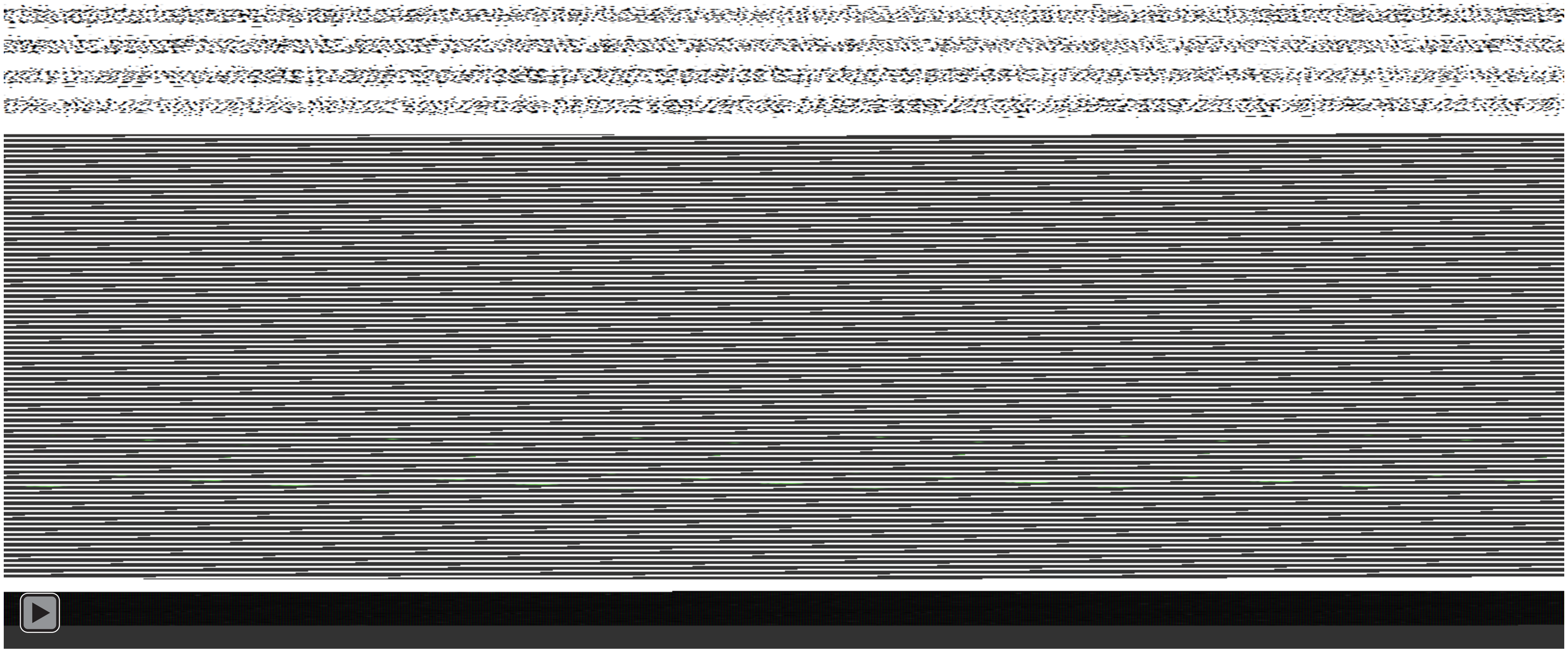

Supplement: qpag036_Supplemental_Files [file qpag036_supplemental_files.zip › Supplemental_Figures_and_Videos.pdf]
